# Effects of fatiguing isometric and isokinetic ankle exercises on postural control while standing on firm and compliant surfaces

**DOI:** 10.1186/1743-0003-9-39

**Published:** 2012-06-14

**Authors:** Etienne J Bisson, Anthony Remaud, Sébastien Boyas, Yves Lajoie, Martin Bilodeau

**Affiliations:** 1 School of Human Kinetics, Faculty of Health Sciences, University of Ottawa, Ottawa, Canada; 2 Aging and Movement Research Laboratory, Bruyère Research Institute, Ottawa, Canada; 3 School of Rehabilitation Sciences, Faculty of Health Sciences, University of Ottawa, Ottawa, Canada

**Keywords:** Neuromuscular fatigue, Posturography, Centre of Pressure, Contraction mode, Plantarflexor muscles

## Abstract

****Background**:**

Fatiguing exercises used across studies to induce alterations in postural control are diverse and may explain the different findings reported. This study aimed to compare the effects of two types of fatiguing plantarflexion exercises on postural control on a firm and a compliant surface. Ten healthy young men (29 ± 4 years) were asked to stand as steadily as possible for 30 s, blindfolded with feet together, on a firm and a compliant surface before and immediately after an isometric and an isokinetic fatiguing exercise.

****Results**:**

Maximal force reduction due to fatigue was found significant but similar between exercises. No significant difference was found between the fatiguing exercises on all Center of Pressure (CoP) parameters. Both fatiguing exercises induced increases in CoP excursion area, CoP variability and CoP velocity in both planes (antero-posterior, mediolateral) on the compliant surface. On the firm surface, both fatiguing exercises only induced increases in CoP variability and CoP velocity in the fatigued plane (antero-posterior).

****Conclusions**:**

Isometric and isokinetic fatiguing exercises, when producing a similar level of force reduction, induce similar decreases in postural control. The effects of fatigue on postural control in healthy young men are more pronounced when standing on a compliant surface, i.e. when proprioceptive information at the ankle is altered.

## **Background**

The ability to perform a multitude of physical activities and most daily living activities requires adequate postural control. In order to maintain postural control, the central nervous system must integrate and (re-)weigh information from the visual, vestibular and somatosensory systems and modulate commands to the neuromuscular system continuously [1]. Not surprisingly, numerous studies [2-11] have demonstrated an impairment in postural control in different postural tasks after neuromuscular fatigue. Since the plantarflexor muscles constitute the main controllers in quiet standing [12], the effect of fatigue of these muscles on postural control have been largely examined. The findings, however, vary significantly possibly due to methodological differences (i.e. fatigue protocols, postural stances used and/or amount of visual information available) across studies. In a recent study [4], we found that an isometric fatiguing exercise (standing on tip-toes until exhaustion) increased Center of Pressure (CoP) excursion area, CoP variability and CoP velocity in both antero-posterior (AP) and medio-lateral (ML) planes. This increase was found independent of the difficulty of the postural task (unipedal, tandem, bipedal), except for a more pronounced effect for the most challenging postural task (i.e. unipedal stance with eyes closed). Our results were consistent with other studies using a similar isometric fatiguing exercise [5,8-11]. In contrast, when the ankle musculature was fatigued with an isokinetic exercise in a different study [2], an increase in CoP velocity was found only in the AP plane, i.e. the plane in which the fatigued plantarflexor muscles act as prime mover muscles. Again, these results were consistent with studies that have used a similar fatiguing exercise [3,6,13]. In addition to the different modes of contraction (isometric vs. isokinetic), other parameters specific to a given fatiguing exercise (intensity, duration) can explain why results vary across studies. Thus, it can be questioned if the impairment of postural control is really influenced by the mode of contraction or if it is only a factor of intensity and/or duration of the fatigue task.

Intense or prolonged exercise inevitably induces neuromuscular fatigue through various mechanisms, i.e., alterations in the activation of the primary motor cortex, reduction of motor unit discharge rates, alterations of excitation-contraction coupling, and slowing of the contractile apparatus [14,15]. Furthermore, muscle fatigue due to intense or prolonged exercise also affects the proprioceptive system [16,17]. This could be explained by the accumulation of metabolites leading to altered muscle spindle function [17], as well as altered central processing of proprioception via group III and IV afferents [16]. However, it has been shown that the mechanisms involved in muscle fatigue are dependent on the exercise performed to fatigue the muscles (task dependency) [18]. Sustained isometric activities have been suggested to induce greater central fatigue compared to concentric intermittent contractions due to greater metabolite by-products accumulation with the limited blood flow, causing in turn an inhibition of the supraspinal descending drive possibly via small-diameter afferents [14,15,19]. However, the impact of these different modes of contraction on postural control has yet to be compared.

Compliant surfaces (such as temper foams and sway-referenced platforms) are commonly used in postural studies to examine the reliance on proprioceptive information in the control of postural tasks [1,20-25]. They are also used in clinical settings to assess balance [26]. By comparing CoP parameters on a firm and a compliant surface, the latter condition has been shown to be more sensitive to balance problems observed with old age [22,25], pathologies such as vestibular disorder [1] and injuries [20,21]. Since a reduction in proprioception and the re-weighting of this information have been proposed to be the main causes of decreased postural control with fatigue [27], one may expect the effect of fatigue to be different on a firm versus a compliant surface. Several studies [7,28-30] have shown an impairment in postural control when standing on a compliant surface (sway-referenced platform) after fatigue. However, only one study [30] compared fatigue-related changes of CoP parameters between a compliant and a firm surface. The authors found an impairment in postural control after ankle, knee and global fatigue. This effect was not greater on a sway-referenced platform. Since the results of this study may be limited because of methodological issues (i.e. duration and number of trials), more research is needed to clarify these findings.

Thus, in an attempt to elucidate factors contributing to the different reports regarding the effect of fatigue on postural control, the objectives of the present study were: a) to compare the effects of isometric and concentric isokinetic fatiguing exercises on postural control during quiet stance, and b) to assess whether the magnitude of these effects are dependent on the amount of proprioceptive information available (i.e., by comparing postural tasks performed on a firm surface and a compliant surface). It was hypothesized that an isometric fatiguing exercise would induce greater alterations in postural control compared to an isokinetic concentric fatiguing exercise, likely due to a greater increase in metabolites concentration [19]. It was further hypothesized that postural control on a compliant surface would be impaired to a greater extent after fatigue compared to a firm surface because standing on a compliant surface requires more proprioceptive information and greater control by the neuromuscular system.

## **Results**

### **Type of fatigue**

#### **MVC**_**IM**_

**Figure 1:**
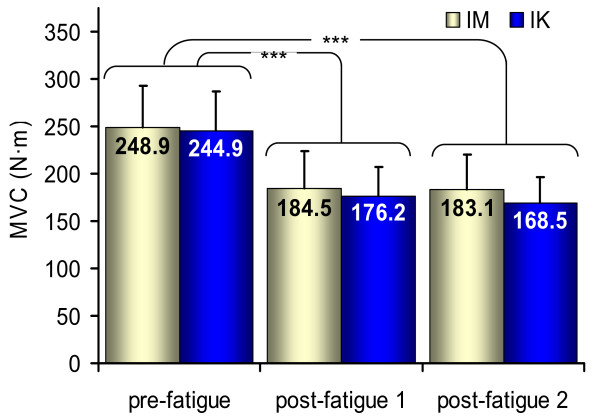
**Changes in torque output.** Mean and standard deviation of MVC_IM_ torque output for each fatiguing exercise. Torque significantly decreased with fatigue (*** p < 0.001), similarly for both types of exercise.

As shown in Figure 1, results on the MVC_IM_ showed a significant main effect of fatigue (F = 94.6, p < 0.001, η_p_^2^ = 0.91). The main effect of type of fatigue (F = 2.6, p = 0.14, η_p_^2^ = 0.22) and the fatigue by type interaction (F = 1.3, p = 0.30, η_p_^2^ = 0.12) were found not significant. MVC_IM_ recorded after each fatiguing exercises (Pooled IM and IK mean values for post-fatigue 1 = 180.3 ± 34.5 N·m and for post-fatigue 2 = 175.8 ± 32.9 N·m) were significantly different from pre-fatigue (Pooled IM and IK mean values = 246.9 ± 42.0 N·m; p < 0.001). The absence of the effect of the type of fatigue suggests that the level of fatigue was similar between the IM and IK fatiguing exercises (mean % of pre-fatigue MVC_IM_ = 73.9 ± 6.7 and 70.9 ± 8.7, respectively).

#### **Time to failure**

Results showed significant main effects of fatigue (F = 11.4, p = 0.01, η_p_^2^ = 0.59), type of fatigue (F = 39.5, p = 0.001, η_p_^2^ = 0.83), and a type by fatigue interaction (F = 17.1, p = 0.003, η_p_^2^ = 0.68). The time to failure of the plantarflexor muscles was significantly longer during the IM session than during the IK session for the first bout of fatiguing exercise (respectively 145.3 ± 34.6 s vs. 54.6 ± 12.1 s, p < 0.001) and for the second bout of fatiguing exercise (respectively 107.0 ± 39.9 s vs. 56.2 ± 13.6 s, p = 0.002). In addition, the time to failure of the first bout of fatiguing exercise was longer compared to the second bout of fatiguing exercise for the IM mode (p = 0.003), but not for the IK mode (p = 0.57).

### **Effect of fatigue on CoP parameters**

For all CoP parameters analyzed, the effect of the type of fatigue (IM vs. IK) and all relevant interactions were found non-significant (p > 0.05). Thus, data from both type of fatiguing exercises were pooled and the subsequent results only describe the effects of fatigue (pre and post-fatigue) and surface (firm, compliant) on the CoP parameters.

#### **CoP excursion area**

**Figure 2:**
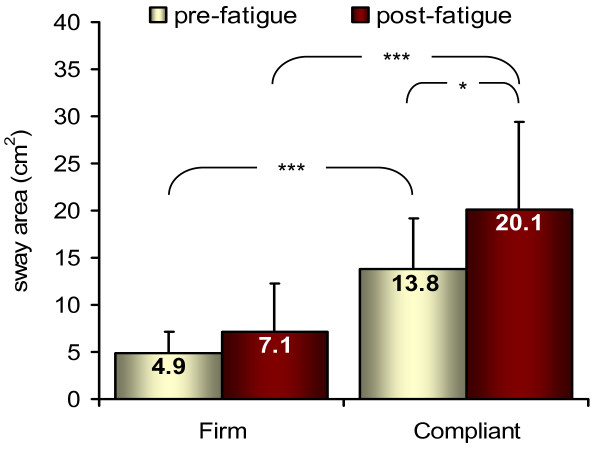
**Changes in CoP excursion area.** Mean and standard deviation of sway area (pooled data from isometric and isokinetic fatiguing exercises). CoP area significantly increased with fatigue when standing on the compliant surface only. * p < 0.05; *** p < 0.001.

Main effects of fatigue (F = 7.9, p = 0.02, η_p_^2^ = 0.47), and surface (F = 91.8, p < 0.001, η_p_^2^ = 0.91) were found significant for the 95% ellipse area parameter. A significant fatigue by surface interaction (F = 12.6, p = 0.006, η_p_^2^ = 0.58) was also found. As shown in Figure 2, CoP excursion area was significantly greater during the compliant condition compared to the firm condition in both pre and post-fatigue (p < 0.001). Also, the effect of fatigue depended on the difficulty of the task (firm vs. compliant surface) since CoP excursion area increased significantly after fatigue when standing on the compliant surface (p = 0.011) but not on the firm surface (p = 0.108).

#### **ML postural control**

**Figure 3:**
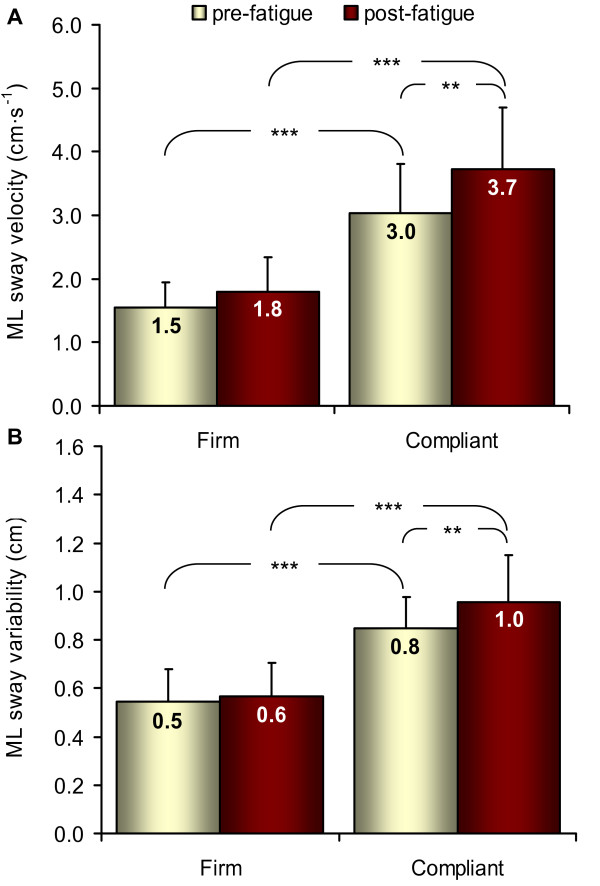
**Changes in ML CoP velocity and variability.** Mean and standard deviation of CoP parameters in ML (pooled data from isometric and isokinetic fatiguing exercises). ML CoP velocity (**A**) and ML CoP variability (**B**) significantly increased with fatigue when standing on the compliant surface only. ** p < 0.01; *** p < 0.001.

Figure 3 shows the results for ML CoP velocity (Figure 3A) and variability (Figure 3B). A main effect of fatigue (F = 12.41, p = 0.006, η_p_^2^ = 0.58) was found significant for ML CoP velocity only. Both CoP velocity and CoP variability showed a significant main effect of surface (velocity: F = 154.9, p < 0.001, η_p_^2^ = 0.95; variability: F = 379.9, p < 0.001, η_p_^2^ = 0.98) and a fatigue by surface interaction (velocity: F = 19.9, p = 0.002, η_p_^2^ = 0.69; variability: F = 12.7, p = 0.006, η_p_^2^ = 0.59). As shown in Figure 3, ML CoP velocity and ML CoP variability was significantly greater during the compliant condition compared to the firm condition in both pre and post-fatigue (p < 0.001). Also, the effect of fatigue depended on the difficulty of the task (firm vs. compliant surface) since ML CoP velocity and ML CoP variability increased significantly after fatigue when standing on the compliant surface (ML CoP velocity: p = 0.003; ML CoP variability: p = 0.003) but not on the firm surface (ML CoP velocity: p = 0.13; ML CoP variability: p = 0.90).

#### **AP postural control**

**Figure 4:**
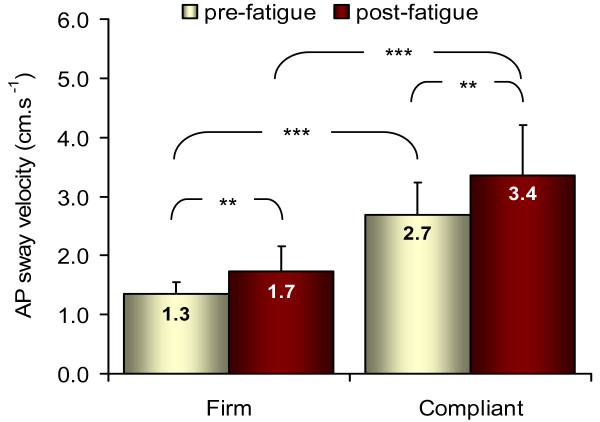
**Changes in AP CoP velocity.** Mean and standard deviation of AP CoP velocity (pooled data from isometric and isokinetic fatiguing exercises). AP CoP velocity significantly increased with fatigue for both surface conditions but appears to be greater when standing on compliant surface. ** p < 0.01; *** p < 0.001.

Both AP CoP velocity and AP CoP variability showed significant main effects of fatigue (velocity: F = 13.5, p = 0.005, η_p_^2^ = 0.60; variability: F = 9.9, p = 0.012, η_p_^2^ = 0.52) and surface (velocity: F = 125.8, p < 0.001, η_p_^2^ = 0.93; variability: F = 193.3, p < 0.001, η_p_^2^ = 0.96). A significant fatigue by surface interaction was found (F = 7.51, p = 0.023, η_p_^2^ = 0.46) for CoP velocity only. AP CoP variability was significantly greater during the compliant condition (0.91 ± 0.07 cm) compared to the firm condition (0.53 ± 0.05 cm) and after fatigue (0.82 ± 0.08 cm) compared to before fatigue (0.67 ± 0.05 cm). As shown in Figure 4, AP CoP velocity was significantly greater during the compliant condition compared to the firm condition in both pre and post-fatigue (p < 0.001). Also, AP CoP velocity increased significantly after fatigue when standing on the compliant surface (p = 0.007) and on the firm surface (p = 0.005). Thus, the significant interaction suggests that AP CoP velocity was increased to a greater extent after fatigue when standing on the compliant compared to the firm surface.

## **Discussion**

This study compared the effects of two types of fatiguing exercises on postural control while standing on a firm and a compliant surface. Our results showed similar amounts of force reduction and decreased postural control between the two fatiguing exercises performed (isometric vs. isokinetic). This refutes our first hypothesis that an isometric fatiguing exercise would induce greater alterations in postural control compared to an isokinetic concentric fatiguing exercise. As expected, all CoP parameters increased when standing on a compliant surface compared to a firm surface. In addition, all five CoP parameters increased with fatigue when standing on a compliant surface whereas only two parameters (those specific to the fatigued AP plane) increased with fatigue when standing on a firm surface. Furthermore, subjects seem to have a greater increase in AP CoP velocity with fatigue when standing on a compliant compared to a firm surface. This confirms our second hypothesis that postural control on a compliant surface with eyes closed is more impaired after fatigue compared to a firm surface.

### **Effects of the type of fatigue**

Both contraction types induced the same amount of force reduction but the isometric fatiguing exercise was of longer duration. This difference could have led to a different effect on postural control. However, no difference in postural control changes with fatigue was observed between contraction modes in this study.

Specific characteristics of the fatiguing exercises could explain the absence of a difference concerning their effect on postural control. For example, the combination of intensity (moderate) and overall duration (relatively short) of both types of fatiguing exercises may have been too similar to lead to differences in fatigue mechanisms (central or peripheral [15]) and consequently on postural control. Time to failure has been reported to be faster with higher-intensity exercises [31]. This, combined with the fact that the transition time between the end of the fatiguing exercises and the post-fatigue measurements may have allowed subjects to partly recover from fatigue, could have reduced our ability to detect a difference in the fatigue effects of both contraction modes used.

However, the fact that fatigue did have a significant impact on postural control, suggests that recovery was not complete. Considering that the force of the plantarflexors was reduced to 75% of the pre-fatigue MVC_IM_ and static postural control requires forces of approximately only 10% MVC [32], it becomes apparent that the reduction in force may not be a major factor explaining the reduction in postural control due to fatigue. Others have suggested that the reduction in postural control due to fatigue may be due to mechanisms other than force-generating capacity, such as altered proprioceptive inputs [8,9,11]. Since the intensity of the fatiguing exercises and their effects on postural control were similar, the proprioceptive deficits might have been equivalent. Our results also suggest that the seemingly different effect of fatigue observed between isometric and isokinetic activities in the literature might be due to methodological factors. Most isometric fatiguing exercises [4,9-11] have typically been of relatively low intensity (lifting body weight) and have been performed directly on the force platform or fairly close to it, leading to minimal recovery between the end of the fatiguing exercise and the start of postural sway acquisition. In contrast, most studies using isokinetic fatiguing exercises [2,6,7] have used a relatively high intensity (50% MVC) and were performed on a dynamometer. Thus, a greater transition time likely occurred and increased the recovery from fatigue prior to the postural testing. Considering the level of intensity (50%) and the use of a dynamometer to induce fatigue, the results (on firm surface) obtained in the present study reflects those reported in studies that used an isokinetic fatiguing exercises. It would be interesting to examine the differences between modes of contraction using a low force fatiguing exercises.

### **Effects of altered proprioceptive information**

In a young healthy population, simple postural tasks can be controlled even when some sensory systems are unavailable or altered [1,25,33-36]. According to the sensory reweighting hypothesis [1,34,36], this is possible since all three sensory systems (visual, vestibular and somatosensory) give redundant information and, “the central nervous system dynamically and selectively adjusts the relative contributions of sensory inputs (i.e. the sensory weights) to maintain upright stance depending not only on the sensory environment, but also on the neuromuscular constraints acting on the subject” [35]. However, these studies showed that postural control decreases considerably when the availability of two sensory systems (somatosensory and vision) are reduced during a standing task [1,25,33-36]. Overall, our results are in line with this sensory reweighting hypothesis [1,34,36]. Subjects were able to maintain their balance, but all CoP parameters tested in this study increased when standing blindfolded on a compliant surface.

However, the original finding in this study is that postural control decreased even more after an ankle fatiguing exercise when the availability of these two sensory systems was reduced. Indeed, both fatiguing exercises increased CoP parameters in both planes (AP and ML) when standing on the compliant surface compared to standing on a firm surface, where CoP parameters increased only in the fatigued plane (AP). In contrast, Dickin and Doan [30] found impairments in postural control due to fatigue (ankle, knee and global fatigue) and type of surface (stable and sway-referenced platform) but no interaction between the two factors. In this latter study, only one trial and one postural parameter (RMS) were used, increasing the variability and potentially decreasing the reliability of the measurements. This may have masked a potential interaction between fatigue and surface type since, according to Ruhe and colleagues [37], at least three trials and parameters in both distance and time-distance domains are needed to accurately characterize postural control.

Vuillerme and colleagues [9] found that vibration of the ankle plantarflexors after fatigue did not show greater effects on postural control compared to fatigue or vibration only. These authors explained their findings by a reduction in sensitivity to the mechanical vibration due to alteration of ankle proprioception (muscle spindles) during fatigue. Since both muscle fatigue and vibration alter the same postural control elements (somatosensory information from the ankle), a combination of the two would not induce greater postural impairments. Conversely, we found in the present study that when subjects were standing on a compliant surface after a fatiguing exercise, their somatosensory information was less reliable potentially due to two distinct mechanisms. First, standing on a compliant surface alters the somatosensory information at the ankle (joint receptors, Golgi tendon organ, muscle spindles) and the sole of the foot (cutaneous mechanoreceptors) in relation to the environment, thus, increasing reliance on other sensory systems (i.e. vestibular) [33]. Second, after fatiguing exercises, the processing of information concerning joint position is altered [38] and force production of the fatigued muscle(s) may be less accurate and more variable [39]. Thus, in the present study, when the kinesthetic references were altered (compliant condition) and muscle position and force sense were impaired (after fatigue), subjects showed greater postural impairments in both planes compared to either condition taken separately. Furthermore, fatiguing the plantarflexor muscles resulted in a decrease in postural control in the ML plane when standing on a compliant but not a firm surface. This is interesting given that postural control in this plane is thought to involve primarly proximal muscles (i.e. hip and back muscles) [40]. However, it has been shown that the contribution of the ankle musculature for quiet standing is still relatively high when feet are less than 8 cm apart [41]. Although the primary muscle group fatigued was the plantarflexors, some of these muscles can also generate torque in the ML plane. Thus, the role of ankle muscles in maintaining postural control in the ML plane with feet together may have been altered after fatigue. To compensate for this, subjects may have re-weighted their sensory inputs to the hip musculature. This strategy may have been effective on a firm surface but not on a compliant surface where the ankle musculature is more solicited.

Although this study was limited to young healthy adults, the impact of these results could be important if found with a more vulnerable population (i.e. older adults, diabetic patients with peripheral neuropathy). These populations, with a potentially less reliable somatosensory system, could be more at risk of falls/injuries following muscle fatigue. Future studies should focus on the impact of muscle fatigue in a population with reduced proprioception.

## **Conclusions**

Previous studies on the effect of ankle fatigue on postural control have shown changes in CoP parameters of different magnitude using different types of fatiguing exercises (different mode of contractions, duration, intensities). The present study aimed to compare the effect of two types of exercise (isometric, isokinetic) with a similar intensity on postural control. We found that isometric and isokinetic fatiguing exercises performed until a similar force reduction induce a similar decrease in postural control. This finding suggests that the decrease in postural control due to muscle fatigue is independent of the mode of contraction performed. Thus, differences between studies are likely due to other factors, such as differences in the force reduction induced by the fatiguing activity, and not due to the type of contraction used. Furthermore, the effects of fatigue on postural control were more pronounced when standing on a compliant surface, i.e. when proprioceptive information at the ankle was altered. This finding supports the literature suggesting that postural control deficits observed after fatigue are largely due to a deficit in proprioception, and suggests that fatigued-related balance impairments leading to falls/injuries could be more frequent in situations where proprioceptive information is reduced (e.g. diabetic or other patient with peripheral neuropathy).

## **Methods**

### **Subjects**

Ten healthy young men (29 ± 4 years, 178.6 ± 7.5 cm, 73.6 ± 9.6 kg) were recruited to participate in this study. Subjects had no neurological problems and no history of falls or ankle injury in the past year. The study was approved by the University of Ottawa and the Bruyère Continuing Care research ethics boards, and written informed consent was obtained from each subject prior to their participation in the study.

### **Procedures**

Subjects took part in two identical sessions, performed one week apart, where postural control was assessed before and immediately after an isometric (IM) or isokinetic (IK) plantarflexion fatiguing exercise. For both sessions (IM session and IK session), postural control was assessed under two surface conditions (firm versus compliant surface), where three 30-s trials of standing as still as possible with feet together and blindfolded (opaque ski goggles) were collected before and after fatigue. This postural task (feet together) without vision was shown to be more sensitive to fatigue on a firm surface compared to the same task with vision [4,8]. Thus, 3 postural trials × 2 types of surface (firm and compliant) × 2 time points (before and after fatigue) for a total of 12 trials were performed per session. A medium density temper foam (41 × 47 × 8 cm; density = 108 kg/m^3^) was used for the compliant surface condition. When standing on this type of foam, subjects were unable to feel the floor surface, thus, proprioceptive information from the feet and ankles was less reliable. Subjects practiced each postural task prior to the pre-fatigue trials. Subjects were asked to position their hands on their hips at the beginning of the trials and were allowed to use them to maintain their balance if necessary.

After completing a first bout of fatiguing exercise (IM or IK depending on session), subjects performed the post-fatigue postural trials in one condition (firm or compliant). Then, after completing the same fatiguing exercise a second time (second bout), subjects performed the post-fatigue postural trials in the other condition (compliant or firm). The fatiguing task was repeated between the two post-fatigue postural conditions in order to ensure the maintenance of an equivalent fatigue state for both conditions. The time elapsed between the end of the fatiguing exercise and the start of the first post-fatigue postural trial for each condition was 40 s on average. The order of the sessions (IM and IK session) and postural conditions (firm, compliant) were counterbalanced between subjects.

### **Fatiguing exercises**

**Figure 5:**
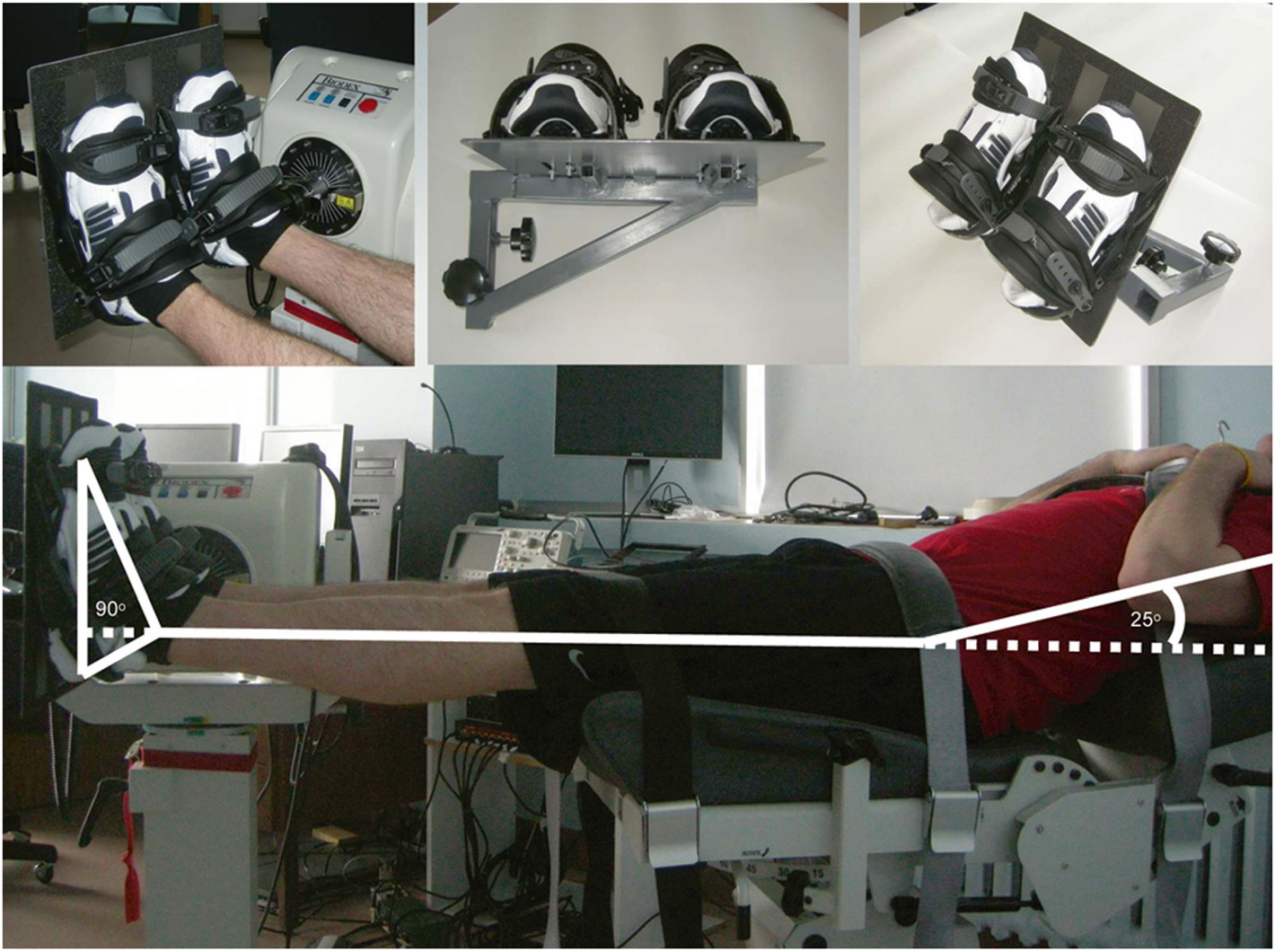
**Experimental setup.** Initial position for the MVCs and the fatiguing exercises. Also depicted is the custom attachment which allowed the simultaneous assessment of both ankles on the Biodex System 3.

Both fatiguing exercises were completed using a Biodex system 3 dynamometer (Shirley, NY, USA) and a custom attachment allowing the production of force using plantarflexor muscles from both ankles simultaneously (Figure 5). During both sessions, subjects were first secured on the dynamometer in the initial position (Figure 5) where legs were horizontal, the trunk elevated to form a 25° angle, and the angle between the legs and the feet fixed at 90°. Subjects performed 10 submaximal isokinetic contractions (30°·s^-1^) and five submaximal isometric contractions of increasing intensities as a warm-up and to be familiarized with the dynamometer. Subsequently, for both IM and IK session, subjects performed three maximal isometric voluntary contractions (MVC_IM_) where the highest peak torque output was considered the subject’s pre-fatigue MVC_IM_. The same procedure was used to calculate the maximal voluntary isokinetic concentric contraction (MVC_IK_) before the IK fatiguing exercise (IK session only).

The fatiguing exercise for the IM session consisted of maintaining 50% of pre-fatigue MVC_IM_ until exhaustion (i.e. subjects unable to maintain the force target for 3 s). Whereas the fatiguing exercise for the IK session consisted of performing continuous maximal concentric IK contractions of the plantarflexors. Angular velocity was set at 30°·s^-1^ with a total range of motion of 35° (from 10° dorsiflexion to 25° plantarflexion, with 0° = initial position). The isokinetic fatiguing exercise ended when the torque decreased below 50% of MVC_IK_ for three consecutive contractions. To avoid any muscle fatigue of dorsiflexor muscles, after each plantarflexion contraction through the 35^o^ range of motion, an experimenter quickly pulled back the attachment to the starting position (10° dorsiflexion) and subjects were instructed to relax during that time. After completion of the first and second bouts of fatiguing exercise for both IM and IK session, subjects were asked to perform a MVC_IM_ (post-fatigue 1, post-fatigue 2) before transferring to the force platform to compare the level of fatigue after both types of exercise. Strong verbal encouragements and visual feedback of the torque output were provided to subjects during the MVC contractions and fatiguing exercises.

### **Data acquisition**

The torque output from the dynamometer was sampled (5000 Hz) via a 1401Plus analog-to-digital board using Spike2 v.7 (CED, Cambridge, UK). CoP data were collected using an AMTI AccuGait force-platform (Watertown, MA, USA) at a 50 Hz sampling rate. The changes in CoP variations associated with fatigue were quantified with three postural variables using BioAnalysis 2.1 software (Watertown, MA, USA): the CoP excursion area (cm^2^) represented by the 95% ellipse area [42], CoP variability (cm) represented by the standard deviation of the CoP, and mean CoP velocity (cm·s^-1^). CoP variability and velocity variables were computed separately for the ML and AP directions. The mean of the three trials for each surface and fatigue conditions was calculated and used for statistical analyses.

### **Data analysis**

A two-way analysis of variance (ANOVA) with repeated measures was used to assess the effects of fatigue (pre-fatigue, post-fatigue 1 and post-fatigue 2) and type of fatigue (IM and IK) on MVC_IM_. A two-way ANOVA with repeated measures was used to assess the effects of fatigue (post-fatigue 1 and post-fatigue 2) and type of fatigue (IM and IK) on time to failure (the time elapsed to reach the fatigue state criterion of each fatiguing exercise). Separate three-way ANOVAs with repeated measures were used to assess the effects of fatigue (pre and post-fatigue), type of fatigue (IM and IK) and type of surface (firm and compliant) for each of the five postural variables (CoP excursion area, ML and AP CoP variability, ML and AP CoP velocity). For all statistical tests, the significance level was set at 0.05. Post hoc analyses were completed when appropriate using Bonferroni adjustments. Normal distribution of the data was confirmed with Kolmogorov-Smirnov tests.

## **Competing interests**

None of the authors have any financial and non-financial competing interests related to this work.

## **Authors’ contributions**

EJB conceptualized the study, carried out the data collection, statistical analyses and drafted the manuscript. AR and SB participated in the design of the study, the data collection, data analyses and the writing of the manuscript. YL participated in the analysis and interpretation of the data, and revised the manuscript. MB participated in the conception and design of the study and revised the manuscript. All authors read and approved the final manuscript.
